# Anger, race, and the neurocognition of threat: attention, inhibition, and error processing during a weapon identification task

**DOI:** 10.1186/s41235-021-00342-w

**Published:** 2021-11-20

**Authors:** Adrian Rivera-Rodriguez, Maxwell Sherwood, Ahren B. Fitzroy, Lisa D. Sanders, Nilanjana Dasgupta

**Affiliations:** 1 Department of Psychological and Brain Sciences, University of Massachusetts Amherst, 135 Hicks Way, Amherst, MA 01003 USA; 2 Neuroscience and Behavior Program, University of Massachusetts Amherst, 230 Stockbridge Road, Amherst, MA 01003 USA; 3 Department of Psychology and Education, Mount Holyoke College, 50 College Street, South Hadley, MA 01075 USA

**Keywords:** Anger, Race, Bias, Threat, EEG, N1, P2, N2, ERN, Pe

## Abstract

This study measured event-related brain potentials (ERPs) to test competing hypotheses regarding the effects of anger and race on early visual processing (N1, P2, and N2) and error recognition (ERN and Pe) during a sequentially primed weapon identification task. The first hypothesis was that anger would impair weapon identification in a biased manner by increasing attention and vigilance to, and decreasing recognition and inhibition of weapon identification errors following, task-irrelevant Black (compared to White) faces. Our competing hypothesis was that anger would facilitate weapon identification by directing attention toward task-relevant stimuli (i.e., objects) and away from task-irrelevant stimuli (i.e., race), and increasing recognition and inhibition of biased errors. Results partially supported the second hypothesis, in that anger increased early attention to faces but minimized attentional processing of race, and did not affect error recognition. Specifically, angry (vs. neutral) participants showed increased N1 to both Black and White faces, ablated P2 race effects, and topographically restricted N2 race effects. Additionally, ERN amplitude was unaffected by emotion, race, or object type. However, Pe amplitude was affected by object type (but not emotion or race), such that Pe amplitude was larger after the misidentification of harmless objects as weapons. Finally, anger slowed overall task performance, especially the correct identification of harmless objects, but did not impact task accuracy. Task performance speed and accuracy were unaffected by the race of the face prime. Implications are discussed.

## Significance statement

The murder of Amadou Diallo, an innocent Black man, on February 4th, 1999 by four plain clothes New York City police officers served as a reminder that racially biased policing continued to plague the United States (U.S.), despite many White Americans’ beliefs that the U.S. had moved past racial injustice. The murder of George Floyd on May 25th, 2020 by Minneapolis police reminds us that racially biased policing remains a systemic issue that continues to kill Black and brown Americans to this day. While racially biased policing takes many different forms, and is influenced by many historical, economic, social, and psychological factors, the present research homes in on two basic psychological effects that may contribute to race-based shooter bias (e.g., a decision to shoot an unarmed Black person). These are the *threat superiority effect* (the propensity to quickly attend to threatening vs. non-threatening stimuli) and the *weapon bias effect* (the tendency to misidentify harmless objects as weapons when paired with Black people). In the current study, we go a step further and examine whether anger impacts the threat superiority effect and weapon bias effect through a neurocognitive lens. Using a sequential priming task, we examine how anger influences the neural correlates of race processing and error recognition, consistent with threat superiority or weapon bias, among a sample of undergraduate students. By identifying social cognitive processes influenced by anger, we hope to inform future research on racially biased decision making relevant to policing.

## Introduction

Rapidly identifying threatening stimuli in one’s environment is pivotal for survival. As such, it is no surprise that research on attentional vigilance consistently shows people have a propensity to attend more quickly to negatively valanced stimuli compared to positive ones (Dijksterhuis & Aarts, 2003; Pratto & John, 1991; Wentura et al., 2000; Williams et al., 1996). This phenomenon, better known as the *threat superiority effect,* has traditionally been examined with evolutionarily relevant threats (e.g., snakes and spiders) (Fox and Damjanovic, 2006; Öhman, 1993; Öhman & Mineka, 2001). However, recent research has found similar effects in response to modern threats (e.g., guns and syringes) that in some cases are stronger than responses to evolutionarily relevant threats (Blanchette, 2006; Fox et al., 2007; Subra et al., 2018). Indeed, our ability to quickly attend to and identify threatening stimuli in contemporary urban life is arguably more important for survival in the twenty-first century than vigilance to spiders or snakes. However, the threat superiority effect can also be maladaptive in certain situations. Consider for example the killing of Amadou Diallo, an unarmed Black man fatally shot by four New York City police officers who misidentified Diallo’s wallet for a gun. In this situation, the threat superiority effect drove the misidentification of a harmless wallet as a threatening weapon; a mistake that cost Diallo, an innocent man, his life.

At the time, questions were raised as to whether Diallo’s race influenced the killing, motivating social psychologists to examine whether racial stereotypes associating Blacks with violence affect visual object processing (Baumann & DeSteno, 2010; Correll et al., 2002, 2006; Payne, 2001). Early research developed laboratory analogs to examine whether race primes (e.g., visual images of Black and White individuals) affected accuracy on speeded weapon detection and revealed a *weapon bias effect* (Payne, 2001). Racial stereotypes linking Black men with threat biased individuals’ responses on the weapon identification task, such that they were more likely to misidentify harmless objects as weapons after seeing Black (compared to White) faces. The weapon bias effect, which has since been replicated in multiple studies (Payne, 2001; Payne et al., 2005; Correll et al., 2002; Correll et al., 2007), has proven invaluable in furthering our understanding of how race and racial stereotypes inappropriately influence perception and judgment in ways that make the threat superiority effect maladaptive. However, other social psychological factors that magnify threat, such as emotions, have not been examined as deeply.

Emotions play a functional role in helping humans navigate social life and influence the degree to which stereotypes guide social judgments (DeSteno et al., 2004; Dasgupta et al., 2009; Bodenhausen et al., 2001). Negative emotions, like anger, are of particular interest in the context of both the threat superiority and weapon bias effects. Anger is known to facilitate automatic responses in threatening situations (Scott, 1980), increasing the likelihood of misidentifying harmless objects as weapons during a weapon detection task (Baumann & Desteno, 2010). Moreover, anger also motivates heuristic processing during social judgment tasks (Bodenhausen et al., 1994), facilitating the activation of racial stereotypes that drive the weapon bias effect (Unkelbach et al., 2008). Together, this research suggests that anger primes threat sensitivity and activates stereotypes, increasing the likelihood that harmless objects will be misidentified as weapons, especially after seeing a Black (compared to a White) face, and may have real-life implications for law enforcement officers.

Alternatively, anger may have a different effect on social judgments because of its link to goals and motivation. Specifically, anger motivates approach behavior, (Carver & Harmon-Jones, 2009; Harmon-Jones, 2019; Harmon-Jones et al., 2003; Peterson et al., 2008; ﻿^2011^; Harmon-Jones, 2004), goal-oriented behavior (Schmitt et al., 2019), and persistence (Lench & Levine, 2008; Seckler et al., 2017). Anger facilitates goal attainment and task performance by focusing attentional and memory resources toward task-relevant stimuli and away from task-irrelevant stimuli (Harmon-Jones, 2019). Thus, it is possible that anger may actually improve performance on a weapon identification task by: (1) focusing attention on task-relevant objects, (2) shunting attention away from task-irrelevant race primes and reducing activation of associated stereotypes, and/or (3) increasing sensitivity to errors consistent with the threat superiority effect (i.e., misidentifying harmless objects as weapons) or errors consistent with the weapon bias effect (i.e., misidentifying harmless objects as weapons after seeing Black faces).

This leads to two competing hypotheses about the possible effects of anger on race and object processing, as well as overall performance, during a weapon identification task. The first hypothesis is based on the links between anger, threat sensitivity, and stereotype activation:

(**H1**) Anger will increase attention and vigilance toward Black (compared to White) faces and decrease sensitivity to errors consistent with threat superiority and weapon bias effects, leading to slower identification of harmless objects (compared to weapons) as well as greater misidentification of harmless objects (compared to weapons) following Black face primes.

The second hypothesis is based on the link between anger and goal attainment motivation:

(**H2**) Anger will improve task performance by decreasing attention and vigilance toward irrelevant face stimuli, increasing attention to relevant object stimuli, and increasing sensitivity to errors consistent with threat superiority and weapon bias effects, leading to faster identification of both weapons and harmless objects as well as less difference in misidentification of harmless objects (compared to weapons) following Black and White face primes.

While a significant amount of research has examined how race processing can influence performance on the weapon identification task (Amodio et al., 2004; Amodio et al., 2008, Correll et al., 2006), very few studies have examined how anger influences weapon identification by modulating attentional orientation and vigilance toward racial cues or sensitivity to errors. In the current study, we induced participants to experience anger or calmness, and recorded electroencephalography (EEG), while they performed a weapon identification task to assess the impact of anger on different aspects of race and object processing. In so doing, we aim to shed light on the impact of anger on specific cognitive processes involved in object identification in order to better inform how emotion and race influence split-second perceptions and decisions that have implications for real-world situations.

In the following sections, we briefly review (1) behavioral evidence demonstrating the weapon bias effect, (2) neural correlates of visual processing associated with attention and vigilance to race and racial stereotypes, and (3) neural processing of errors consistent with the weapon bias effect. We also review existing emotion studies to support our competing hypotheses regarding the possible effects of anger on neural correlates involved in visual and error processing during a weapon identification task.

### Behavioral evidence in support of the weapon bias effect

The effects of racial cues on weapon identification have been an important research topic among social psychologists for the last two decades. In a landmark study, Payne (2001) created a sequential priming task to demonstrate that the race of primes (i.e., Black and White faces) influences the magnitude of the threat superiority effect. Specifically, Payne showed that participants were faster to identify guns, and slower to identify tools, after exposure to Black compared to White faces. Furthermore, Black (but not White) face primes increased misidentification of harmless tools as weapons when participants were pressured to respond quickly. Using the process dissociation procedure (Jacoby, 1991)—a method of quantifying the extent to which performance on various cognitive tasks is driven by automatic vs. controlled processes—Payne (2001) revealed that the weapon bias effect was driven by the automatic associations linking Black men with handguns.

In a related paradigm, Correll et al. (2002) examined the effect of race on participants’ decisions to “shoot” armed targets or “not shoot” unarmed targets in a simulated first-person shooter task. Like Payne’s (2001) weapon identification task, Correll’s shooter task illuminated racial biases in response times, such that participants’ decision to shoot Black armed targets was faster than their decision to shoot White armed targets. Conversely, when the target was unarmed, participants took longer in their decision to not shoot when the target was Black compared to White. Similar to Payne (2001), pressures to respond quickly during the shooter bias task led to a significant increase in racially biased mistakes (i.e., shooting an unarmed Black target). In exploratory analyses, Correll found that knowledge of cultural stereotypes associating Blacks with danger (not necessarily personal endorsement of such stereotypes) predicted stronger racial bias on the task.

While the above-mentioned studies vary in detail, the conclusions regarding the influence of race on the threat superiority effect are consistent. First, the presence of a racial cue (whether presented as a prime, or simultaneously with the target stimulus) systematically biases the time it takes to correctly identify objects as either harmless (e.g., tool), or dangerous (e.g., gun), such that stereotype congruent pairings (i.e., Black—gun, White—tool) are processed faster than stereotype incongruent pairings (i.e., Black—tool, White—gun). Second, racially biased responses result from individuals’ knowledge of *societal* stereotypes associating Blacks with danger, regardless of their personal endorsement of such stereotypes. Third, external pressure to respond quickly compromises cognitive control processes necessary to overcome bias, allowing automatic stereotypes associating Black Americans with threat to contaminate participants’ judgments of objects as dangerous even when they are not, resulting in higher error rates for stereotypically incongruent pairings (Black—tool) compared to congruent pairings (Black—gun). Together, these findings hint at the cognitive processes of stereotype activation and subsequent failure to control that leads to racially biased responding on weapon identification tasks.

### Neural indices of visual attention and cognitive control

The current study used EEG techniques to examine event-related brain potentials (ERP) associated with attention allocation (N1 and P2) and controlled inhibition (N2) processes in response to race stimuli on a weapon detection task (Mangun & Hillyard, 1987, 1991). The visually evoked N1 is a negative-going ERP component peaking approximately 150–200 ms after event onset. The visually evoked P2 is a positive-going ERP component peaking approximately 200 ms after event onset. It is well documented that while the visual N1 and P2 covary, they are distinct components (Luck & Kappenman, 2011) associated with early attention allocation (N1; Mangun & Hillyard, 1987, 1991) and sustained attention/feature selection (P2; Anllo-Vento et al., 1998; Hillyard & Münte, 1984) processes respectively.

The visually evoked N1 component is understood to be modulated by selective attention to visual stimuli, (Luck & Kappenman, 2011). N1 amplitude reflects attentional allocation based on spatial location (Mangun, 1995; Mangun & Hillyard, 1987, 1991), as well as low-level characteristics (e.g., luminance; Johannes et al., 1995) of visual stimuli, with larger amplitudes elicited over both posterior and anterior scalp regions by attended stimuli (Eason, 1981; Harter & Aine, 1984; Hillyard & Münte, 1984; Hillyard et al., 1998). Furthermore, it has been shown that N1 amplitudes are sensitive to attentional focus (i.e., focusing attention completely on a single task vs. dividing attention across multiple tasks). For example, Mangun and Hillyard (1990) showed that N1 amplitudes were larger when participants were instructed to provide 100% of their attention to detect target stimuli at a single spatial location, compared to when instructed to divide their attention to detect target stimuli across multiple spatial locations. Researchers interpret this attentional modulation of the visual N1 component as evidence of ‘sensory gain control’, a term referring to the neurocognitive mechanisms involved in increasing or decreasing attention toward tasks relevant and irrelevant cues (also referred to as ‘amplification’; Hillyard et al., 1998).

The anterior visually evoked P2 component is understood to reflect attention to stimuli features such as color, orientation, and size (Anllo-Vento et al., 1998; Hillyard & Münte, 1984; Luck & Hillyard, 1994), with larger amplitudes elicited by attention to task-relevant stimuli features. For example, research by Hillyard and Münte (1984) examined N1 and P2 sensitivity to stimulus color at attended vs. unattended spatial locations. While N1 amplitudes were unaffected by stimulus color at both attended and unattended spatial regions, P2 amplitudes were larger in response to attended stimuli color, but only at attended spatial regions. At unattended spatial regions, P2 amplitude did not differ in response to attended vs. unattended color. These findings suggest that attention to stimuli features (indexed by the visually evoked P2 component) is contingent on, but functionally different from, early attention allocation processes (indexed by visually evoked N1 components) (Hillyard & Münte, 1984; Luck & Kappenman, 2011), and suggests that the visually evoked P2 component may also index sustained attention.

The visually evoked N2 is a negative-going ERP component peaking approximately 200–350 ms after event onset. Like the N1 and P2, the N2 is associated with attentional allocation (Eimer, 1993; Hickey et al., 2006; Luck & Hillyard, 1994), but has also been linked to research on novelty and mismatch (Folstein & Van Petten, 2008; Folstein et al., 2008; Patel & Azzam 2005﻿). Anteriorly distributed N2 components have also been linked to more strategic processes like cognitive control (Folstein & Van Petten, 2008; Ritter et al., 1979). Several studies have found the anterior N2 to reflect response inhibition, with larger N2 amplitudes elicited by the inhibition of a planned response during a go/no-go task (Bruin & Wijers, 2002; Pfefferbaum et al., 1985; Jodo & Kayama, 1992; Falkensten et al., 1999), and correct responses to incongruent noise conditions during the Eriksen Flanker Task (Bartholow et al., 2005; Yeung et al., 2004). Importantly, evidence suggests that N2 increases elicited by response inhibition during go/no-go tasks are modulated by task difficulty, such that larger amplitudes are elicited when participants are pressured to respond quickly (Jodo & Kayama, 1992). Finally, research by Falkenstein et al. (1999) and Kopp et al., (1996) found significant associations between N2 amplitude and successful response inhibition during a go/no-go task, providing further evidence in support of the link between the N2 component and cognitive control processes related to inhibition.

The visual N1, P2, and N2 components have also been examined in the context of race processing among White college students (Ito & Urland, 2003, 2005; Ito et al., 2004). Research on N1 amplitudes during race processing has been mixed. One study examining automatic attention allocation during a face encoding task found the N1 to be sensitive to race, such that Black faces elicited larger amplitudes compared to White faces. This seems to suggest that participants showed greater automatic attention allocation to Black faces compared to White faces (Ito & Urland, 2003). However, this effect of race on the N1 component was not replicated during tasks where faces were presented as task-irrelevant stimuli (Ito & Urland, 2005). One interpretation of these conflicting findings is that N1 amplitude sensitivity to race may depend on whether race is a relevant cue or not for the task at hand.

Research on the P2 and race processing has been more consistent, with larger amplitudes elicited by Black compared to White faces (Ito & Urland, 2003, 2005). The N2 component has also been shown to be sensitive to race, with larger amplitudes elicited by White faces compared to Black faces, both during passive viewing (Ito & Urland, 2003; Ito et al., 2004), and when presented as irrelevant stimuli (Ito & Urland, 2003). Consistent with research on sustained attention and inhibition processes outlined above, Ito et al. (2004, see also Ito & Urland, 2005) interpret these findings to indicate greater activation of sustained attentional processes, also referred to as vigilance by Ito & Urland (2005), in response to unfamiliar racial out-group members (i.e., Black faces) compared to familiar racial in-group members (i.e., White faces), and greater activation of cognitive control processes in response to racial in-group, compared to out-group, members.

Researchers have also examined the visual N1, P2, and N2 components during a shooter bias task (Correll et al., 2006). While N1 amplitudes were only shown to be differentially sensitive to objects (i.e., larger amplitudes in response to weapons vs. harmless objects), P2 and N2 amplitudes were both differentially sensitive to objects (i.e., larger P2 and smaller N2 amplitude for weapons vs. harmless objects) and the race of the target (i.e., larger P2 and smaller N2 amplitude for Black compared to White men). One interpretation of these findings, consistent with the automatic attention allocation, sustained attention, and inhibition frameworks outlined above, is that participants automatically attended more to weapons than harmless objects, were most vigilant during trials where Black men held weapons, and exerted the most cognitive control on trials where White men held harmless objects. Furthermore, these biases in race and object processing were associated with racially biased performance on the shooter bias task, such that greater activation of attention, and less activation of inhibition processes, in response to Black (vs. White) targets predicted more incorrect decisions to shoot unarmed Black men compared to unarmed White men (Correll et al., 2006).

While these findings were seminal in laying the groundwork toward understanding the neurocognitive mechanisms that contribute to racially biased responses during a weapon identification task, they are not without shortcomings. For example, in the research by Correll et al. (2006), race processing during the shooter bias task is confounded with object processing, due to the simultaneous presentation of both race and object stimuli during each trial of the task. Thus, it is unclear whether the race effects on P2 and N2 amplitudes, as well as the lack of race effects on N1 amplitude, were purely in response to racial cues, or whether they were driven by the simultaneous presentation of stereotypically congruent (e.g., Black men—guns) and incongruent (e.g., Black men—harmless objects) race—object pairings.

This confound can be addressed by examining visual processing components like the N1, P2, and N2 within a sequential priming paradigm like the weapon identification task that decouples responses to dangerous and harmless objects from responses to racial cues like faces (Payne, 2001). To our knowledge, no published study has investigated whether and how automatic shunting of selective attention and inhibition processes varies as a function of racial cues to influence subsequent object processing.

### Neural processing of errors consistent with the weapon bias effect

EEG techniques have also been used to examine ERPs associated with error processing, specifically the Error-Related Negativity (ERN) and Error-Related Positivity (Pe), during a weapon identification task. Both the ERN and the Pe are believed to propagate from the anterior cingulate cortex (ACC) (Herrmann et al., 2004), a neural structure interconnected with both limbic and prefrontal areas of the brain, and functionally linked to the processing and regulation of motor, cognitive, and affective information (Bush et al., 2000).

The ERN, a well-documented error processing component, is a negative-going evoked potential that peaks 50–100 ms after a response that is larger for incorrect vs. correct responses (Yeung et al., 2004). Further, research has found the amplitude of ERNs to incorrect responses to be affected by the significance of errors (Hajcak et al., 2005; Hajcak & Foti, 2008), with larger ERN amplitudes typically associated with more costly errors. ERN amplitude has also been linked to motivation (Hajcak & Foti, 2008; Pailing & Segalowitz, 2004; Potts, 2011). For example, research by Potts (2011) examined ERN amplitudes during a Flanker task where performance was linked to monetary rewards. Results showed that ERN amplitudes were largest after errors made during trials where incorrect responses resulted in monetary loss, suggesting that ERN amplitudes were sensitive to participant’s motivations to maximize monetary gain.

The ERN has also been examined in association with error processing during Payne’s weapon identification task (Amodio et al., 2004, 2008). These studies found that among individuals motivated to respond without bias on the task, racially biased errors (e.g., misidentification of a harmless object following a Black prime) elicit larger ERN amplitudes compared to non-biased errors (e.g., misidentification of a weapon following a Black prime). Additionally, larger ERN amplitudes following racially biased errors predicted greater behavioral accuracy on the weapon identification task. In line with previous research on the ERN and motivation, Amodio et al. (2008) found evidence to suggest that ERN amplitudes sensitivity to racially biased errors was modulated by individual differences in participants’ motivation to control prejudice. Specifically, racially biased errors elicited larger ERN amplitudes among individuals who indicated that they were highly, and personally, motivated to control prejudice, compared to individuals who were less personally motivated. Together, these findings suggest that automatic error detection processes (as indexed by ERN amplitude following incorrect responses) play an important role in weapon identification task performance, especially among individuals personally motivated to respond without prejudice. However, it is still unclear how automatic recognition of racially biased errors leads to better performance, as this would suggest that some controlled processes must be activated to overcome automatic race and object processes that influence task performance.

One component that may explain the link between ERN and better task performance on the weapon identification task is the Error-Related Positivity (Pe) component. The Pe is related to but functionally distinct from the ERN (for a review see Overbeek et al., 2005), and is associated with controlled error processing, and cognitive re-evaluation processes required for response inhibition (Donchin et al., 1988; Herrmann et al., 2004; Leuthold & Sommer, 1999). The Pe has also been suggested to reflect conscious awareness of errors during an antisaccade task (Nieuwenhuis et al., 2001), such that larger Pe amplitudes were elicited by perceived, compared to unperceived, errors. While the Pe has not been examined within the context of the weapon identification task, we suggest that Pe amplitude may also be sensitive to errors consistent with both threat superiority and weapon bias effects. Given the documented links between the Pe, conscious awareness of errors, and cognitive re-evaluation following errors on cognitive tasks, Pe sensitivity to errors elicited by object or racial biases may indicate the extent to which participants are aware that they are making biased errors during the weapon identification task, and reflect adaptation during the task through controlled inhibition of biased responses. For this reason, we argue that examination of Pe, along with ERN, within a weapon identification task is critical for furthering our understanding of how error processing can impact cognitive control to improve weapon identification accuracy.

### The effects of anger on attention, inhibition, and error recognition

Anger has been shown to strengthen the threat superiority effect, increasing vigilance to weapons and misidentification of harmless objects during a weapon detection task (Baumann & Desteno, 2010). Anger has also been shown to magnify implicit bias toward out-group members, presumably because of the association of anger with intergroup conflict and competition (Dasgupta et al., 2009; DeSteno et al., 2004). Such magnification of bias may also be related to anger-induced increases in confidence (Clore et al., 2001), or anger-induced heuristic processing when making social judgments (Bodenhausen et al., 1994). For these reasons, one might predict that anger would exacerbate errors consistent with threat superiority and weapon bias effects on a weapon detection task by increasing attention and vigilance to Black (compared to White) faces, and decreasing the recognition and inhibition of biased errors.

However, previous research by Unkelbach et al. (2008) examined the effect of affect (i.e., anger, positive mood, neutral mood) on racial bias during a shooter bias task that used Muslim and White targets. Racially biased responses (i.e., shooting unarmed Muslim targets) were elicited among neutral and positive feeling participants. Interestingly, angry participants did not show racially biased responses; instead, they showed greater propensity to shoot all targets regardless of race compared to positive or neutral participants. In other words, anger did not increase racially biased responding during a shooter bias task, which conflicts with the research summarized earlier.

The lack of race bias effects on shooter bias task accuracy among angry participants in the work by Unkelbach et al. (2008) may be partially explained by research showing that anger functions as a goal-oriented motivator, that can positively impact cognitive performance by suppressing task-irrelevant information (Carver & Harmon-Jones, 2009; Harmon-Jones, 2019; Harmon-Jones et al., 2003; Peterson et al., 2011; Harmon-Jones, 2004; Schmitt et al., 2019). This explanation, however, fails to address why angry participants were more likely to shoot *all* targets compared to neutral and happy participants. Thus, a conceptual replication of these conflicting findings is warranted, to distinguish between the two competing hypotheses (H1, H2) stated above.

### The current study

In the current study, we examine the effects of anger on visual attention, inhibition, and error recognition processes during a sequential priming weapon identification task, to better understand the neurocognitive mechanisms by which anger influences the threat superiority and weapon bias effects. To test our competing hypotheses, we examined the effect of anger on ERPs associated with early attention allocation (N1), sustained attention (i.e., vigilance; P2) and response inhibition (N2) during race prime processing, as well as automatic error recognition (ERN) and subsequent controlled error recognition and task re-evaluation processes (Pe) following threat assessment, in a variation of Payne’s (2001) weapon identification task. Thus, this study examined neurocognitive mechanisms across the entire time course of the weapon identification task, from the moment that faces varying in race appeared as primes, followed by harmless or threatening objects, followed by participants’ response.

We propose two competing sets of predictions. If anger activates stereotypes as some research suggests (H1: e.g., DeSteno et al., 2004; Dasgupta et al., 2009; Bodenhausen et al., 1994), we predict that angry (vs. neutral) participants will show (1) greater threat superiority and weapon bias effects as indexed by accuracy and response time during the weapon identification task; (2) increased attention to, and reduced inhibitory processing of, Black compared to White faces, as indexed by larger N1 and P2 amplitudes and smaller N2 amplitudes; and (3) reduced recognition and re-evaluation of errors after making racially biased errors or misidentifying harmless objects as weapons, as indexed by smaller ERN and Pe amplitudes. Alternatively, if anger motivates goal attainment by focusing attentional and memory resources to task-relevant stimuli, while simultaneously ignoring task-irrelevant stimuli (H2: e.g., Carver & Harmon-Jones, 2009; Harmon-Jones, 2004, 2019; Harmon-Jones et al., 2003; Peterson et al., 2011; Schmitt et al., 2019; Unkelbach et al., 2008), we predict (1) reduced threat superiority and weapon bias effects as indexed by accuracy and response time during the weapon identification task; (2) reduced attention to, and increased inhibitory processing of, both Black and White face primes, as indexed by smaller N1 and P2 amplitudes and larger N2 amplitudes; and (3) increased recognition and re-evaluation of any type of error, regardless of whether they are racially stereotypic errors or misidentification of harmless objects as weapons, as indexed by larger ERN and Pe amplitudes.

## Method

### Participants

Data were collected from 131 University of Massachusetts Amherst students. Participants were all non-Black [consistent with sampling methods used in Payne (2001)], right-handed participants between the ages of 18 and 35. All participants were screened for the use of psychoactive medication within the last six months. Of the 131 participants, data from 28 were excluded from analysis for failing the emotion manipulation check. Behavioral data from the remaining 103 participants (93 White, 7 Asian, 3 Latino/Hispanic; 72 Female; 50 anger condition, 53 neutral condition) were included in the behavioral analyses examining accuracy and response time during the task. ERP data from 76 (35 anger condition, 41 neutral condition) of those 103 participants were included in ERP analyses. ERP data from the remaining 27 participants were excluded from ERP analyses due to making fewer than 3 errors in at least one condition (which is insufficient for calculating error-related ERPs; *n* = 13), having more than 80% of responses be of one type (either weapon or harmless objects) such that correctness was more closely related to the correspondence between object type and response bias than a decision made on each trial (*n* = 10), or showing excessive artifacts in ERP averages (*n* = 4).

### Procedure

Upon entering the study, participants were randomly assigned to either the anger or neutral emotion condition, and were led to believe they would be participating in two unrelated tasks: a decision-making task (i.e., the weapon identification task) and an autobiographical memory task (i.e., the emotion induction task). Participants in both conditions first completed a practice version of the weapon identification task. Next, participants were asked to engage in an alleged autobiographical writing task, which was actually designed to induce an angry or neutral emotional state. This emotion induction task consisted of two separate 5-min writing blocks during which participants wrote about a time in their life when they were very angry (anger condition), or a description of their apartment or dorm room layout (neutral condition). After writing for 5 min, participants were asked to complete block 1 of the weapon identification task. They then returned to the emotion induction task and continued writing where they had left off for another 5 min. After the second writing period, participants completed block 2 of the weapon identification task. After completing block 2, participants completed a self-report measure of their emotional state while they were engaged in the autobiographical writing task and provided demographic information. Finally, participants were debriefed regarding the true nature of the study, and given the option to watch a short positive film to counteract lingering negative emotions.

### Weapon identification task

**Fig. 1:**
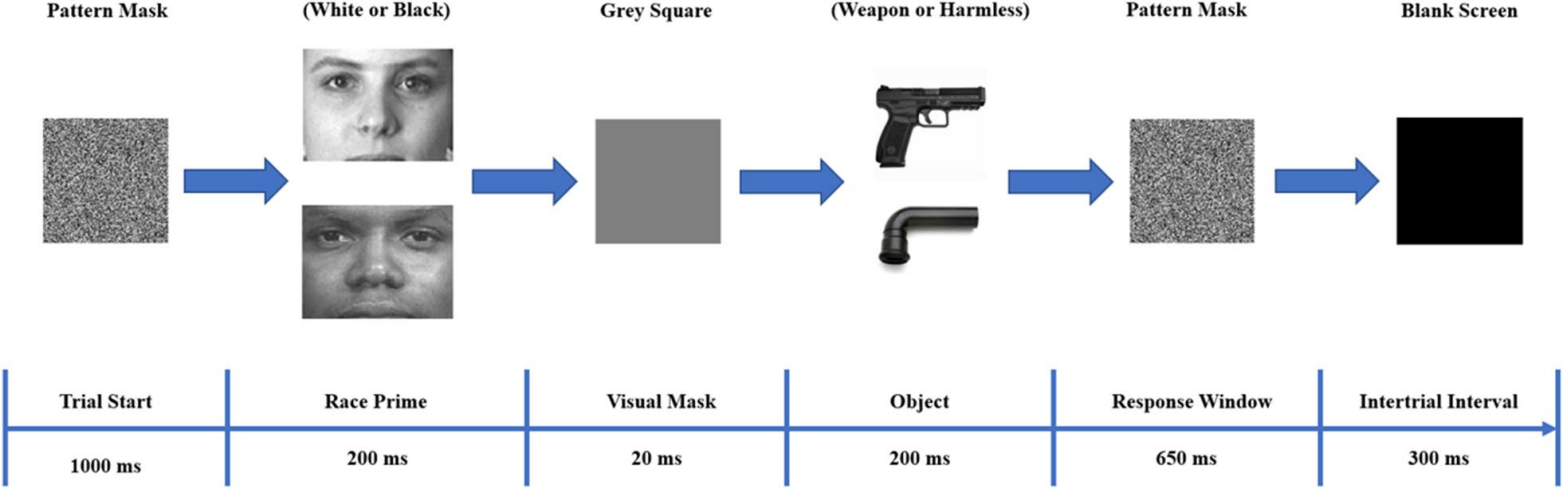
Example trial from the weapon identification task used in the current study, adapted from Payne (2001)

The weapon identification task used in the current study was adapted from Payne’s weapon identification task (2001), with modifications to better control for stimulus factors and make the task more difficult (Fig. 1). We created a more difficult version of the original task to ensure participants would make enough errors to reveal a reliable ERN. Prior to starting the task, participants were told that “several images, including patterns, faces, and objects, would be flashed on screen”. Participants were further instructed to “focus on the objects, and to quickly press one of two buttons to categorize the object as either harmless, or a weapon”. The association between the two buttons (one corresponding to their left hand and the other to their right) and the two categories (i.e., weapon or harmless object) were counterbalanced across participants.

Each trial started with a pattern mask (white noise) displayed on a computer monitor for 1000 ms. Next, either a Black or a White face displayed for 200 ms. Following the face prime, a gray square was presented on the screen for 20 ms followed by either a weapon or a harmless object for another 200 ms. Following the object, the pattern mask was displayed for another 650 ms, during which a button press was made to indicate whether the object was a weapon or a harmless object (response window). Finally, there was a blank screen of 300 ms after the response window (intertrial interval). Participant responses that were made either in the response window or the intertrial interval were recorded and included in analysis. If participants responded during the intertrial interval, or if they failed to respond at all, they received the following onscreen visual warning for 1000 ms in white text on a black background: “You did not respond fast enough on that trial. Remember to respond as quickly as possible!”.

To increase the difficulty of our weapon identification task compared to that of Payne (2001), we used a larger set of faces and objects and more closely matched the weapons and harmless objects (see “Appendix”). Specifically, we included 12 faces (6 Black faces, 6 White faces), 30 weapons (6 handguns, 6 assault rifles, 6 knifes, 6 bombs, 6 pairs of nunchucks), and 30 harmless objects (6 L-shaped pipes, 6 windshield ice scrapers, 6 pens, 6 bundles of firewood, 6 pairs of candles). The faces were identical to those used in previous studies of implicit bias (Nosek et al., 2007). The object images were initially found through a Google Images search and then modified for this study. Unlike the original weapon identification task, we qualitatively matched each weapon image to a harmless object image by shape, orientation, and brightness. Thus, participants were less able to rely on simple visual features to differentiate between weapons and harmless objects. We included a gray square between the face and the object on each trial so that any similarities in basic visual features between a category of faces (Black, White) and a category of objects (weapons, harmless objects) would be less likely to affect responses. An example weapon identification trial can be seen in Fig. 1. Each block of the weapon identification task consisted of 240 trials, with 60 trials for each Race Prime (Black vs. White) × Object Type (weapon vs. harmless) combination. The order in which trials were presented was randomized for each block, and across all participants.

### Emotion induction and manipulation check

The emotion induction for participants in the anger group, and the control task for participants in the neutral group, was disguised as an autobiographical writing task and has been used in several studies to successfully induce specific emotions in participants (Dasgupta et al., 2009; DeSteno et al., 2004; Mills & D’Mello, 2014). In the current study, participants in the anger condition were given the following prompt: “Please take a moment to remember a time that you were **Very Angry**. When you have recalled this memory, focus on it so that you have a vivid impression of the events involved. Take a minute to experience the feelings that you felt at that time. Once you have done this, please describe the memory in as much detail as you can.” Participants in the neutral condition were given the following prompt: “Please take a moment to remember your **Dorm Room or Apartment**. When you have recalled this memory, focus on it so that you have a vivid picture of the room(s). Once you have done this, please describe your room(s) in as much detail as you possibly can.”.

At the end of the study, participants completed a self-report measure of their emotional state while engaged in the autobiographical writing task. Participants rated how angry, calm, afraid, sad, mad, relaxed, disgusted, irritated, scared, fearful, peaceful, and happy they felt on 7-point Likert scales (1 = Not at all, 7 = Very Much). Ratings for angry, mad, and irritated items were averaged to create a composite anger index (Chronbach’s alpha = 0.97). Ratings of calm, relaxed, peaceful were averaged to create a composite neutral index (Chronbach’s alpha = 0.97). We also created a composite fear rating by averaging across afraid, scared, fearful ratings (Chronbach’s alpha = 0.94). Happiness, sadness, and disgust remained single items.

### EEG data acquisition and processing

EEG was recorded with 128-Channel HydroCel™ Geodesic Sensor Nets (EGI, Eugene OR). Electrodes were soaked in a water and potassium chloride solution to maintain impedances below 60 kΩs. Data were initially recorded with a 250 Hz sampling rate, a bandpass of 0.01–100 Hz, and referenced to the central-midline electrode (Cz in 10/20 system). The 128 electrode locations included sites directly above and below both eyes to detect blinks and vertical eye movements, and at the outer canthi of each eye to detect horizontal eye movements. All data were re-referenced offline to the average of the left and right mastoid recordings.

Individual subject data were processed using EEGLAB (Delorme & Makeig, 2004) and ERPLAB (Lopez-Calderon & Luck, 2014) toolboxes in MATLAB. We first applied a 60 Hz Parks-McClellan notch filter. Independent Component Analysis (ICA) was applied to continuous EEG data to remove artifacts related to blinks where possible. In participants with a 1st ICA component that was consistent with the timing and topography of blinks, this component was excluded before all other components were recombined, and a high pass filter of 0.1 Hz was applied. Prime-locked epochs were extracted from continuous EEG 100 ms before to 500 ms after the onset of the race prime. Response-locked epochs were extracted from continuous EEG 100 ms before to 500 ms after correct and incorrect responses. Epochs with remaining blink or other artifacts were rejected using a combination of algorithms and manual rejection. Prime-locked epochs were averaged separately by participant for each electrode and Race (Black, White) by Object (weapon, harmless) condition, and baseline corrected to the average amplitude during the 100 ms before race prime onset. Response-locked epochs were averaged separately by participant for each electrode and Race by Object by Correctness (incorrect, correct) condition, and baseline corrected to the average amplitude during the 100 ms before the response.

### ERP analysis

For both the prime-locked and response-locked ERPs, data from 84 electrodes were included in analyses. To treat topographic distribution as a two-dimensional construct, data from 7 proximal electrodes were averaged together within each of 12 scalp regions, organized as a 3 (lateral) by 4 (anterior-to-posterior position) grid (see Fig. 4 for details).

#### Prime-locked ERPs

Mean amplitude measurements were made in three time windows selected based on visual waveform inspection, time-locked to the onset of the race prime. Early measurements captured the waveforms during presentation of a race prime (N1: 100–150 ms), and as a race prime was replaced by a gray square (P2: 150–225 ms), and then an object (N2: 225–300 ms). Omnibus ANOVAs on mean amplitude in these time windows included the between-subjects factor Emotion (anger, neutral), and within-subjects factors Race prime (Black, White), lateral electrode position (LMR: left, medial, right) and anterior-to-posterior electrode position (ACP: anterior, anterior-central, posterior-central, posterior).^1^

#### Response-locked ERPs

Mean amplitude measurements were made in three time windows selected based on visual waveform inspection for response-locked ERPs. Measurements captured the waveforms immediately after a response in the typical Error-Related Negativity (ERN) window (25–75 ms), in a late ERN window (75–125 ms),^2^ and in a 300–400 ms time window that captured the error-related positivity (Pe). Omnibus ANOVAs conducted separately on mean amplitude in each time window included the between-subjects factor Emotion (anger, neutral), and within-subjects factors Race prime (Black, White), Object (weapon, harmless), Correctness (correct, incorrect), lateral electrode position (LMR), and anterior-to-posterior electrode position (ACP). Significant interactions of Correctness and electrode position factors were followed up by ANOVAs on data from electrode regions where the effect of Correctness was largest, to test hypotheses concerning the relationship between Emotion and Race prime in making and evaluating rapid weapon-or-harmless-object decisions.

## Results

### Sample size justification and sensitivity analysis

Previous research found effects of race on N1, P2, N2 (Correll et al., 2006; Ito and Urland, 2003, 2005; Ito et al. 2004), and ERN (Amodio et al., 2004, 2008) with sample sizes as small as 16 and as large as 73. Across these studies, the average sample size was 37.7. Because our study includes a between-subjects’ factor (i.e., Emotion), we aimed to obtain a sample size that was at least twice as large as this average (behavioral analysis: *N* = 103; ERP analysis: *N* = 76).

We also conducted a sensitivity analysis using G*Power 3.1 (Faul et al, 2007) to calculate the smallest effect size we could detect for the two types of analyses (*t* tests and mixed method ANOVAs) reported in this study. Effect sizes were calculated based on sample size (behavioral analysis: *N* = 103; ERP analysis: *N* = 76), power (0.80), an alpha of 0.05, between-subject levels (2), within-subject levels (2), and the lowest observed pairwise correlation among our within-subject factors (0.23). Note that we did not include electrode positions factors (LMR, ACP) in our sensitivity analyses because these factors were not central to our hypothesis. Results from these analyses are presented in Table 1.

**Table 1:** Sensitivity analyses: smallest detectable effect size based on analysis parameters

	*N*	Between-subjects groups	Within-subject measurements	Power	*α*	Lowest observed pairwise correlation	Effect size
*Behavioral analyses*							
Paired-sample *t* tests	103	2	2	.80	.05	NA	.28
Mixed Method (Between-Within) ANOVA							
Main Effect of Emotion	103	2	2	.80	.05	.23	.21
Main Effect of for within-subject variables	103	2	2	.80	.05	.23	.17
Between-Subject × Within-Subject Interactions	103	2	2	.80	.05	.23	.17
*ERP analyses*							
Paired-sample *t* tests	76	2	2	.80	.05	NA	.32
Mixed Method (Between-Within) ANOVA							
Main Effect of Emotion	76	2	2	.80	.05	.23	.25
Main Effect of for Within-Subject Variables	76	2	2	.80	.05	.23	.20
Between-Subject × Within-Subject Interactions	76	2	2	.80	.05	.23	.20

Sensitivity analyses are reported for paired-sample *t* tests and mixed method ANOVAs, separately for behavioral and ERP analyses. We used the lowest observed pairwise relationship among within-subject factors to obtain conservative estimates of the lowest effect size our analyses would be able to detect. Effect sizes for *t* test are reported in Cohen's *d*, effect sizes for ANOVAs are reported in Cohen's *f*

### Emotion manipulation check

A qualitative analysis of written responses to the emotion induction prompts indicated that all participants followed the writing prompt instructions. To ensure that the difference in participants’ emotional state between the two conditions was primarily due to anger and not mixed emotions, we ensured that self-reported emotions from participants in the anger condition had an anger composite rating that was at least 1 point higher than other emotion ratings. Similarly, we ensured that participants in the neutral condition had composite calm ratings that were at least 1 point greater than other emotion ratings. Data from participants who did not meet these criteria (*n* = 28) were excluded from further analysis.

As expected, independent sample *t* tests showed that participants in the anger condition reported feeling angrier (*M* = 5.28, SD = 1.09) during the weapon identification task than participants in the neutral condition (*M* = 1.34, SD = 1.04; *t*(101) = 18.81, *p* < 0.001). Conversely, participants in the neutral condition reported feeling more calm (*M* = 6.06, SD = 0.91) during the weapon identification task than participants in the anger condition *(M* = 2.24, SD = 1.21; *t*(101) = − 18.26, *p* < 0.001). Descriptive means for self-reported emotions (i.e., composite anger, composite calm, happy, sad, disgust, and composite fear) are reported separately by emotion condition in Table 2.

**Table 2:** Mean self-reported emotions, separately by emotion condition

Emotions	Condition			
	Anger		Neutral	
	Mean	SD	Mean	SD
Anger	5.28	1.09	1.34	1.04
Calm	2.24	1.21	6.06	0.91
Happy	1.39	0.67	5.00	1.39
Sad	3.51	1.93	1.58	1.06
Disgusted	3.84	1.89	1.23	0.82
Fearful	1.68	1.17	1.08	0.27

Mean self-reported emotion scores and standard deviations (SD) reported separately by emotion condition. Anger, calm and fearful emotions are composite scores

### Behavioral results

#### Response accuracy

**Fig. 2:**
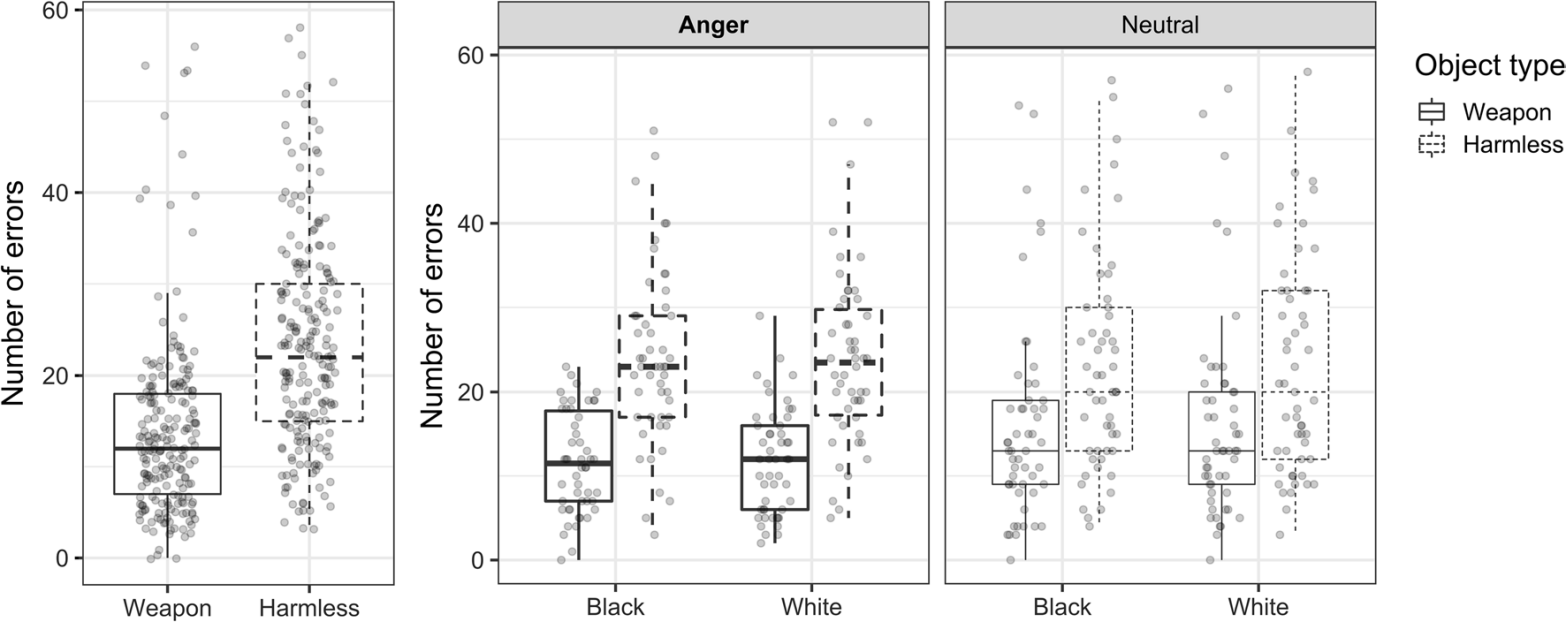
Boxplots detailing the number of errors as a function of object type, both averaged across emotion condition and race prime (left) and plotted separately by emotion condition and race prime (right)

A mixed three-way ANOVA (Emotion × Race × Object) was conducted on the number of errors made on the weapon identification task. Consistent with the threat superiority effect, participants were more likely to misidentify harmless objects (*M*_errors_ = 23.56, SE = 1.14) than weapons (*M*_errors_ = 13.92, SE = 0.93; main effect of Object: *F*(1, 101) = 34.45, *p* < 0.001, *d* = 0.90), suggesting a significant weapon bias. However, unlike previous research, there was no evidence that the race of primes affected accuracy (main effect of Race: *F*(1, 101) = 0.002, *p* = 0.967, *d* < 0.01) or weapon bias (Race × Object: *F*(1, 101) = 0.329, *p* = 0.567, *d* = 0.02). Likewise, anger did not affect accuracy (main effect of Emotion: *F*(1, 101) = 1.85, *p* = 0.177, *d* = 0.07) or weapon bias (Emotion × Object: *F*(1, 99) = 2.67, *p* = 0.106, *d* = 0.29), and emotion and race did not have interactive effects on accuracy or weapon bias (Emotion × Race: *F*(1, 101) = 0.018, *p* = 0.829, *d* = 0.004; Emotion × Race × Object: *F*(1, 101) = 0.02, *p* = 0.888, *d* = 0.004). In other words, neither the race of face primes nor the induction of anger magnified weapon bias. Average numbers of errors across Emotion condition, Race and Object type are displayed in Fig. 2.

#### Response latency

A mixed four-way ANOVA (Emotion × Race × Object × Correctness) was conducted on log transformed response times (log RT), though means are reported here as raw RTs for ease of understanding. Consistent with past work (Dijksterhuis & Aarts, 2003; Pratto & John, 1991; Wentura et al., 2000; Williams et al., 1996), correct responses took longer than incorrect responses (main effect of Correctness: *F*(1, 99) = 129.724, *p* < 0.001, *d* = 0.29). A significant main effect of Object was also found (*F*(1, 99) = 69.52, *p* < 0.001, *d* = 0.13), such that participants were slower to react to harmless objects (*M* = 418.23, SE = 1.43) than weapons (*M* = 402.18, SE = 1.33). These main effects were qualified by a significant Object × Correctness interaction (*F*(1, 99) = 4.13, *p* = 0.045, *d* = 0.18). Follow-up analyses indicated that RTs were consistent with the threat superiority effect, such that participants were significantly slower to correctly identify harmless objects (*M* = 449.62, SE = 2.08) than weapons (*M* = 404.39, SE = 1.51; *t*(201) = 4.57, *p* < 0.001, *d* = 0.32). On incorrect trials, participants were on average faster to misidentify harmless objects as weapons (*M* = 386.84, SE = 1.73) than to misidentify weapons as harmless objects (*M* = 399.97, SE = 2.19), though this difference in RT did not reach significance (*t*(201) = − 0.64, *p* = 0.52, *d* = − 0.05).

Emotion also influenced response time (main effect of Emotion: *F(*1, 99*)* = 4.195, *p* = 0.043, *d* = 0.36), such that participants in the anger condition were slower to respond (*M* = 425.47, SE = 11.29) than those in the neutral condition (*M* = 395.82, SE = 10.96). This main effect of emotion was qualified by a significant Emotion × Object × Correctness interaction (*F*(1, 99) = 4.39, *p* = 0.039, *d* = 0.19). Follow-up analyses disaggregating this interaction by Emotion revealed a significant Object × Accuracy interaction in the anger condition (*F*(1, 48) = 55.55, *p* < 0.001, *d* = 0.55), but not the neutral condition (*F*(1, 51) = 1.13, *p* = 0.973, *d* < 0.01). Specifically, in the anger condition, participants were significantly slower to correctly identify harmless objects (*M* = 473.69, SE = 2.02) than to correctly identify weapons (*M* = 410.21, SE = 1.83; *t*(97) = 13.75, *p* < 0.001, *d* = 1.37). Participants in the anger condition were also faster to misidentify harmless objects as weapons (*M* = 395.73, SE = 2.38) than to misidentify weapons as harmless objects (*M* = 422.25, SE = 2.78), (*t*(97) = 13.75, *p* < 0.001, *d* = − 0.42).

**Fig. 3:**
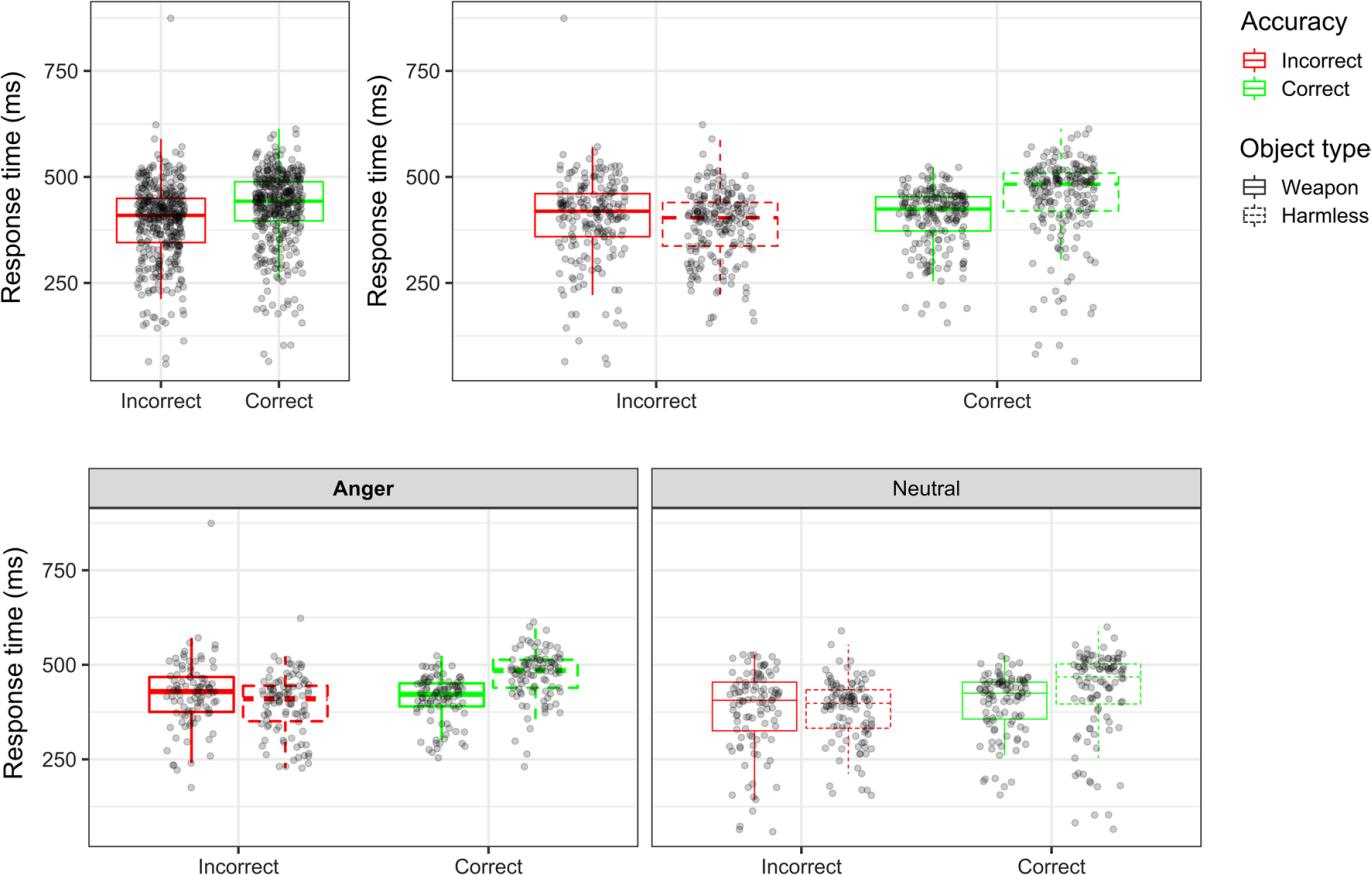
Boxplots detailing raw RTs as a function of response correctness, averaged across both emotion condition and object type (top left), separately by object type (top right), and separately by emotion condition and object type (bottom)

Similar to response accuracy, and contrary to our hypotheses, race had no effect on response times (main effect of Race: *F*(1, 99) = 0.06, *p* = 0.800, *d* < 0.01; all interactions involving race, *p* > 0.25, statistics reported in supplemental information^3^). Average RTs as a function of emotion condition, object type, and correctness are displayed in Fig. 3.

### Neural responses to race primes during the weapon identification task (prime-locked ERPs)

**Fig. 4:**
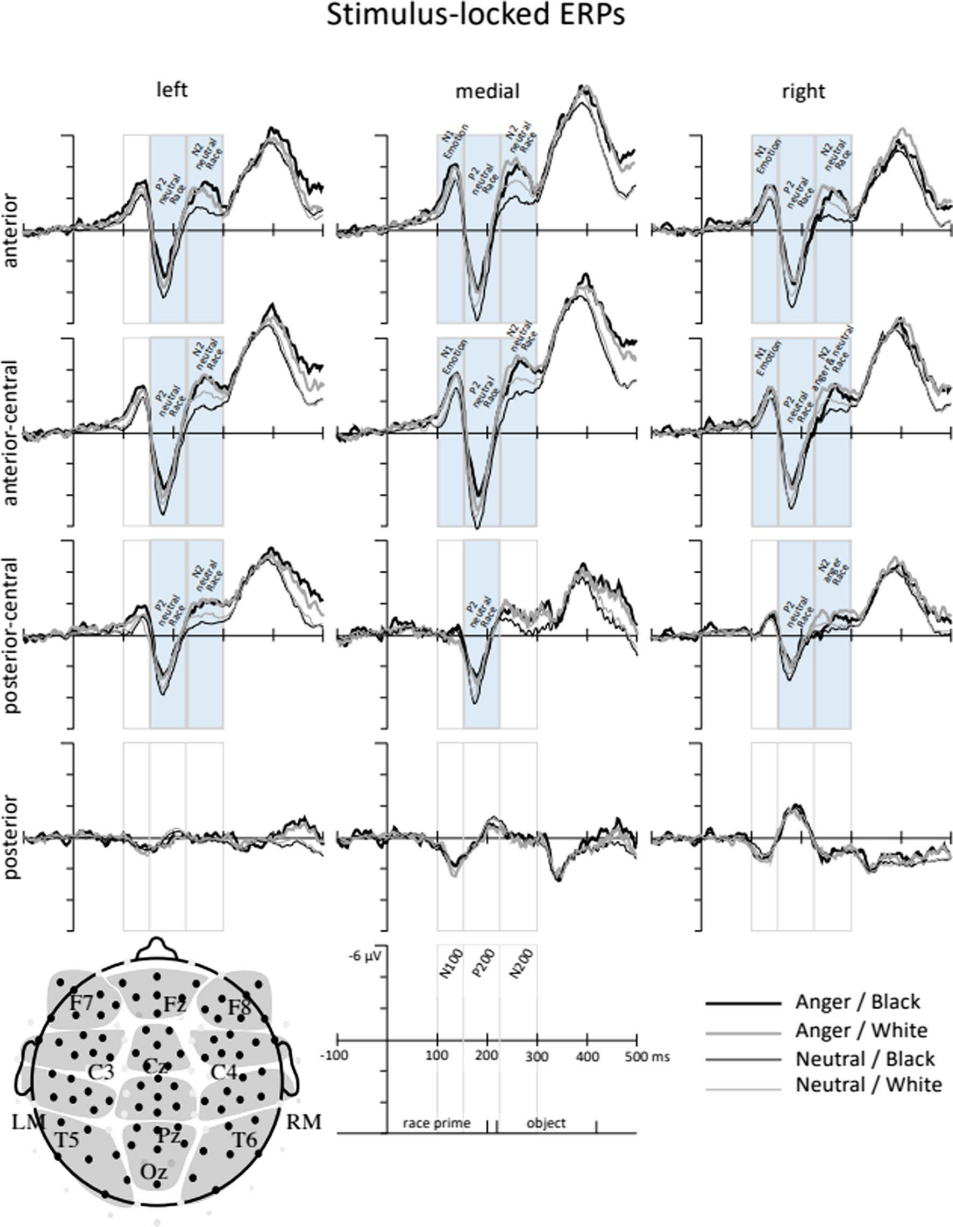
ERP waveforms time-locked to the onset of the race prime. The timing of the ERP measurement windows (N1: 100–150 ms, P2: 150–225 ms, and N2: 225–300 ms) and appearance of the race prime (0–200 ms) and object (220–420 ms) are shown on the scale. Emotion condition is distinguished by line thickness (anger = thicker, neutral = thinner); Race prime is distinguished by line shading (Black = darker, White = lighter). Faces elicited a larger N1 in the anger condition than in the neutral condition. In the neutral emotion condition only, Black faces elicited a larger P2 than White faces over anterior and anterior-central regions. In the neutral condition, White faces elicited a larger N2 than Black faces over medial and right anterior regions. Effect significance over each scalp region is indicated with a filled rectangle

To examine whether anger and race influenced the visual processing of race primes, a four-way omnibus ANOVA (Emotion × Race × LMR × ACP) was run separately for N1, P2, and N2 components as dependent variables. In cases where the omnibus ANOVA yielded significant interactions between electrode position factors (i.e., LMR: left, medial, right; ACP: anterior, anterior-central, posterior-central, posterior) and factors central to our hypotheses (i.e., Emotion, Race), follow-up analyses were conducted over scalp regions where the effects of emotion and/or race were largest. Prime-locked ERP waveforms depicting the effect of emotion and race of prime on N1, P2, and N2 amplitudes can be found in Fig. 4.

#### N1 (100–150 ms)

To examine whether anger and race influenced N1 amplitude, a four-way Omnibus ANOVA (Emotion × Race × LMR × ACP) was run on mean ERP amplitudes 100–150 ms after race prime onset. Race primes elicited a larger N1 in the anger condition (*M* = − 1.00, SE = 0.02) than in the neutral condition (*M* = − 0.59, SE = 0.02; main effect of Emotion: *F*(1, 74) = 5.74, *p* = 0.019, *d* = 0.29). Although the effect of Emotion was evident across multiple electrode regions (as shown in Fig. 4), a significant Emotion × LMR × ACP interaction (*F*(6, 444) = 3.47, *p* = 0.016, *d* = 0.20) suggested that the strength of the Emotion effect on N1 amplitude differed across electrode positions. Follow-up analyses disaggregated this interaction by laterality (LMR electrode regions) and found significant Emotion × ACP interactions at medial (*F*(3, 222) = 3.81, *p* = 0.011, *d* = 0.35) and right (*F*(3, 222) = 5.03, *p* = 0.012, *d* = 0.35) regions. The N1 effect at the left region was nonsignificant (*F*(3, 222) = 2.10, *p* = 0.131, *d* = 0.20). Significant Emotion × ACP interactions at medial and right lateralization were further broken down by anterior-to-posterior electrode position. At the medial region, a significant effect of Emotion was found at anterior (*t*(294) = − 3.82, *p* < 0.001, *d* = − 0.44) and anterior-central regions (*t*(276) = − 3.99, *p* < 0.001, *d* = − 0.46). At the right region, a significant effect of Emotion was also found at anterior (*t*(262) = − 2.40, *p* = 0.017, *d* = − 0.28) and anterior-central (*t*(292) = − 3.33, *p* < 0.001, *d* = − 0.38) regions. While the effect of Emotion on N1 amplitude was significant anteriorly (anterior and anterior-central) at both medial and right lateralization, effect sizes suggest that the effect of emotion was strongest at medial (as compared to right) regions of the scalp (Fig. 4, earliest marked time window). Mean N1 amplitudes by Emotion condition at medial anterior, medial anterior-central, right anterior, and right-anterior-central are reported in Table 3.

**Table 3:** Mean N1 amplitude by Emotion

	Anger		Neutral	
	Mean	SD	Mean	SD
Mid				
Anterior	− 2.77	1.87	− 1.95	1.87
Anterior-central	− 2.47	2.14	− 1.54	1.85
Right				
Anterior	− 1.81	2.09	− 1.66	1.59
Anterior-central	− 1.29	1.64	− 1.05	1.55

Mean N1 amplitudes and standard deviations (SD) reported separately by Emotion, across electrode positions where the effect of Race was significant: medial, anterior, medial anterior-central, right anterior, and right anterior-central

Because we were interested in whether the effect of Emotion on N1 amplitude was influenced by Race, we conducted an additional targeted Emotion × Race ANOVA over medial anterior and medial anterior-central regions where the Emotion effect sizes were largest. This analysis did not yield any evidence to suggest that N1 amplitude, or the effect of Emotion on N1 amplitude, was moderated by Race at either medial anterior (main effect of Race: *F*(1, 74) = 0.11, *p* = 0.739, *d* = 0.04; Emotion × Race: *F*(1, 74) = 1.19, *p* = 0.278, *d* = 0.11), or medial anterior-central (main effect of Race: *F*(1, 74) = 0.00, *p* = 0.968, *d* < 0.01; Emotion × Race: *F*(1, 74) = 0.09, *p* = 0.754, *d* = 0.04) regions.

#### P2 (150–225 ms)

To examine whether anger and race influenced P2 amplitude, a four-way omnibus ANOVA (Emotion × Race × LMR × ACP) was run on mean amplitude 150–225 ms after race prime onset. Marginal interactions between electrode factors and both Emotion and Race (Emotion × ACP: *F*(3, 222) = 2.84, *p* = 0.077, *d* = 0.17; Race × ACP: *F*(3, 222) = 2.99, *p* = 0.054, *d* = 0.48; Emotion × Race × LMR: *F*(1, 148) = 3.13, *p* = 0.057, *d* = 0.47) suggested the possibility of Emotion and Race effects at specific scalp locations. Given that Emotion × Race effects were predicted a priori, this motivated us to run follow-up three-way ANOVAs (Race × LMR × ACP) separately by emotion.

In the anger condition, Race did not affect P2 amplitude (Race × LMR × ACP: *F*(6, 204) = 1.23, *p* = 0.299, *d* = 0.04; Race × LMR: *F*(2, 68) = 2.93, *p* = 0.066, *d* = 0.05; Race × ACP: *F*(3, 102) = 0.28, *p* = 0.718, *d* = 0.03). However, in the neutral condition, a significant Race × ACP interaction (*F*(3, 120) = 4.40,* p* = 0.031, *d* = 0.11) suggested that P2 amplitude differed as a function of race at specific anterior-to-posterior electrode positions (Fig. 4). Disaggregating this interaction by ACP yielded significant effects of race over anterior (*t*(245) = 3.90, *p* < 0.001, *d* = 0.25), anterior-central (*t*(245) = 3.24, *p* = 0.001, *d* = 0.21), and posterior-central (*t*(245) = 2.07, *p* = 0.040, *d* = 0.13) regions, such that larger P2 amplitudes were elicited by Black (compared to White) faces. The effect of race was not significant at posterior regions of the scalp (t(245) = − 0.72, *p* = 0.470, *d* = − 0.05). In sum, these results suggest that P2 amplitudes were sensitive to race primes among participants who were emotionally neutral, but not among participants who were induced to feel angry. Mean P2 amplitudes for both anger and neutral conditions at anterior, anterior-central, and posterior-central regions are reported in Table 4 and shown in Fig. 4 (second marked time window).

**Table 4:** Mean P2 amplitude by Emotion and Race

	Anger				Neutral			
	Black		White		Black		White	
	Mean	SD	Mean	SD	Mean	SD	Mean	SD
Anterior	*0.97*	*3.46*	*0.40*	*2.73*	**2.14**	**2.49**	**1.63**	**2.19**
Anterior-central	*1.21*	*4.02*	*1.18*	*3.10*	**2.40**	**2.66**	**2.01**	**2.34**
Posterior-central	*0.81*	*3.67*	*0.86*	*2.94*	**1.65**	**2.36**	**1.43**	**2.18**

Mean P2 amplitudes and standard deviations (SD) reported separately by Emotion and Race at anterior, anterior-central and posterior-central regions. Bold numbers represent descriptive statistics for significant effects of Race, italicized numbers represent descriptive statistics for non-significant effects of Race

#### N2 (225–300 ms)

To examine whether anger and race influenced N2 amplitude, a four-way omnibus ANOVA (Emotion × Race × LMR × ACP) was run on mean EEG amplitude 225–300 ms after race prime. A main effect of Race indicated smaller N2 amplitudes in response to Black, compared to White, face primes (main effect of Race: *F*(1, 74) = 5.94, *p* = 0.017, d = 0.14) (see Fig. 4). Significant interactions among Race, Emotion, and electrode factors (Race × ACP: *F*(3, 222) = 14.24, *p* < 0.001, *d* < 0.01; Race × LMR × ACP: *F*(6, 444) = 5.69, *p* < 0.001, *d* = 0.05; Emotion × Race × LMR: *F*(2, 148) = 4.02, *p* = 0.028, *d* = 0.05) suggested that the effects of Race and Emotion on N2 amplitude differed across scalp location. This motivated us to run follow-up four-way ANOVAs (Race × LMR × ACP) separately by emotion.

In the anger condition, a significant Race × LMR × ACP interaction (*F*(6, 204) = 2.72, *p* = 0.014, *d* = 0.06) suggested that the effect of Race on N2 amplitude varied across scalp locations. Follow-up analyses separated by LMR regions revealed significant Race × ACP interactions at medial (*F*(3, 102) = 4.87, *p* = 0.012, *d* = 0.11) and right regions (*F*(3, 102) = 5.07, *p* = 0.009, *d* = 0.13) only; no effect was evident in the left region (*F*(3, 102) = 0.98, *p* = 0.404, *d* = 0.05). Within each ACP region, the effect of Race was significant over right anterior-central scalp regions (*t*(69) = 2.00, *p* = 0.049, *d* = 0.24), and marginally significant at right posterior-central scalp regions (*t*(69) = 1.83, *p* = 0.071, *d* = 0.22) only. The effect of Race was not significant over right anterior, right posterior, and any medial scalp regions (*ps* > 0.10). In sum, among participants induced to feel angry, Black faces elicited smaller N2 amplitudes than White faces over a constrained region of right central scalp (see Table 5).

**Table 5:** Mean N2 amplitude by Emotion and Race

	Anger				Neutral			
	Black		White		Black		White	
	Mean	SD	Mean	SD	Mean	SD	Mean	SD
Left								
Anterior	− *2.11*	*3.10*	− *1.98*	*2.59*	**−** **1.14**	**2.97**	**−** **1.81**	**3.18**
Anterior-central	− *3.35*	*3.71*	− *2.81*	*3.07*	**−** **1.29**	**3.09**	**−** **2.12**	**2.93**
Posterior-central	− *1.71*	*3.51*	− *1.82*	*2.48*	**−** **0.67**	**2.64**	**−** **1.21**	**2.29**
Mid								
Anterior	− *2.89*	*3.78*	− *3.50*	*3.48*	**−** **1.58**	**3.93**	**−** **2.61**	**4.13**
Anterior-central	− *3.36*	*3.84*	− *3.89*	*3.75*	**−** **2.06**	**4.06**	**−** **2.95**	**3.8**
Right								
Anterior	− *1.45*	*2.53*	− *1.73*	*2.67*	**−** **0.64**	**3.01**	**−** **1.24**	**3.14**
Anterior-central	**−** **1.72**	**3.02**	**−** **2.44**	**2.93**	**−** **1.05**	**3.31**	**−** **1.68**	**3.14**
Posterior-central	**−** **0.64**	**2.64**	**−** **1.33**	**2.79**	− 0*.32*	*2.37*	−0 *.62*	*2.17*

Mean N2 amplitudes and standard deviations (SD) reported separately by Emotion and Race, across electrode positions: left, medial, right (LMR) and anterior, anterior-central, and posterior-central regions. Bold numbers represent descriptive statistics for significant effects of Race, italicized numbers represent descriptive statistics for non-significant effects of race

In the Neutral condition, a significant Race × LMR × ACP interaction also suggested that the effect of Race on N2 amplitude varied across scalp locations (*F*(6, 240) = 3.83, *p* = 0.004, *d* = 0.03). Follow-up analyses revealed significant Race × ACP interactions at left (*F*(3, 120) = 7.94, *p* = 0.002, *d* = 0.13), medial (*F*(3, 120) = 13.50, *p* < 0.001, *d* = 0.16), and right (*F*(3, 120) = 10.76, *p* < 0.001, *d* = 0.13) regions of the scalp. Looking along the anterior-to-posterior axis over left scalp regions yielded significant effects of Race over anterior (*t*(81) = 2.68, *p* = 0.008, *d* = 0.30), anterior-central (*t*(81) = 3.83, *p* < 0.001, *d* = 0.42) and posterior-central (*t*(81) = 2.71, *p* = 0.008, *d* = 0.30) regions. Over medial scalp regions, significant effects of Race were found at anterior (*t*(81) = 3.86, *p* < 0.001, *d* = 0.43) and anterior-central (*t*(81) = 3.37, *p* = 0.002, *d* = 0.37) regions, but not posterior-central or posterior regions (*p*s < 0.10). Finally, over right scalp regions, significant effects of Race were found at anterior (*t*(81) = 2.84, *p* = 0.006, *d* = 0.31) and anterior-central regions (*t*(81) = 3.12, *p* = 0.002, *d* = 0.35), but not at posterior-central or posterior regions (*p*s < .10). In sum, while Black (compared to White) faces elicited smaller N2 amplitudes participants in both the anger and neutral conditions, this effect of race was much more broadly distributed over the scalp in participants induced to feel neutral. Mean N2 amplitudes for both anger and neutral conditions at all significant electrode locations are reported in Table 5, and shown in Fig. 4 (latest marked time window).

### Neural processing of errors during the weapon identification task (response-locked ERPs)

#### Error-related negativity (ERN)

**Fig. 5:**
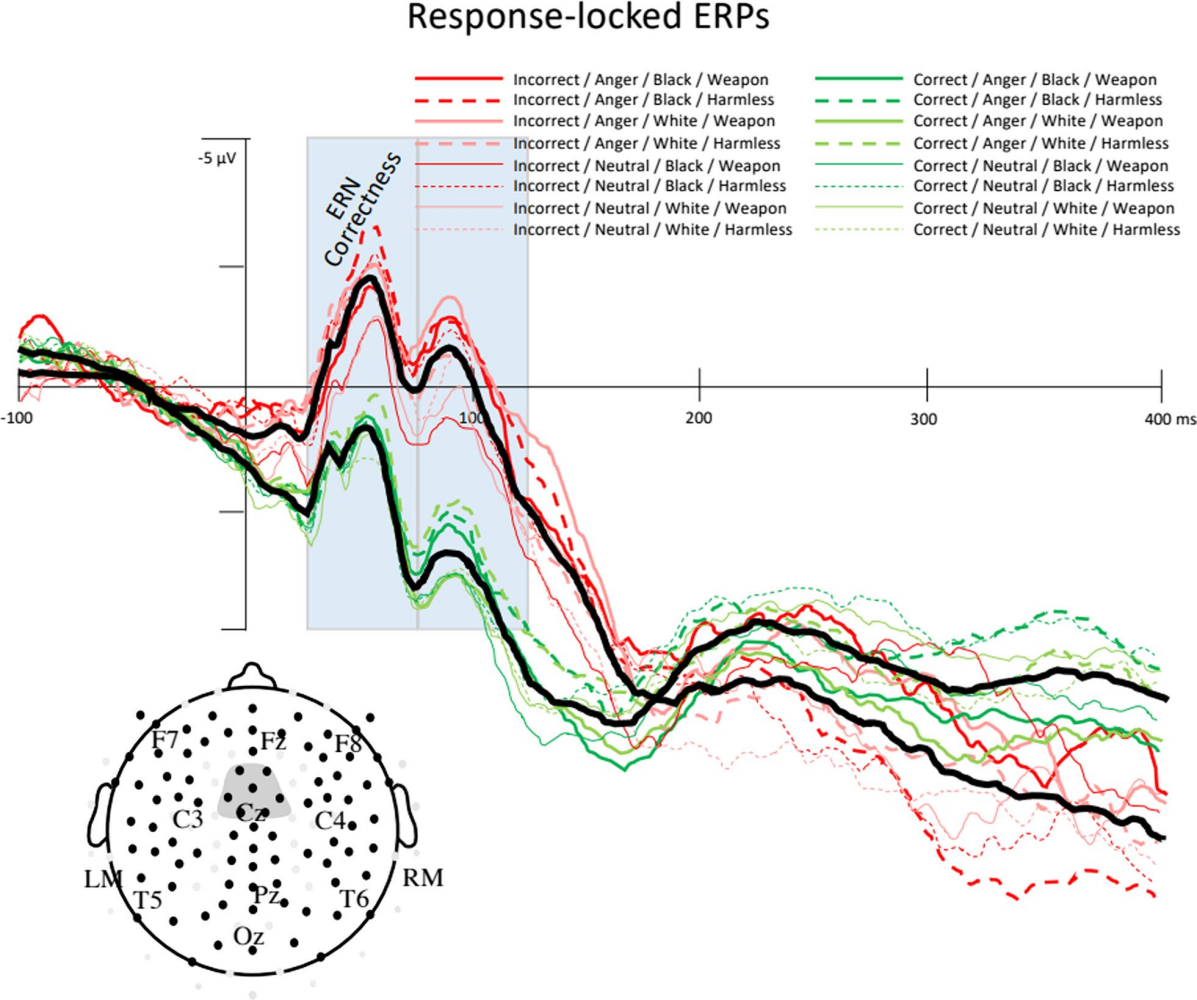
ERP waveforms time-locked to a response at medial anterior-central electrodes only. Correctness is distinguished by line color (incorrect = red, correct = green); Emotion condition is distinguished by line thickness (anger = thicker, neutral = thinner); Race prime is distinguished by line shading (Black = darker, White = lighter); Object type is distinguished by line type (weapon = solid, harmless = dashed). Solid black lines show waveforms averaged across all Correct conditions and all incorrect conditions. Incorrect responses elicited a larger negativity than correct responses in the ERN time window (25–75 ms after response) that carried over into the subsequent time window (75–125 ms after response), with no evidence of modulation by Emotion, Race prime, or Object type

**Fig. 6:**
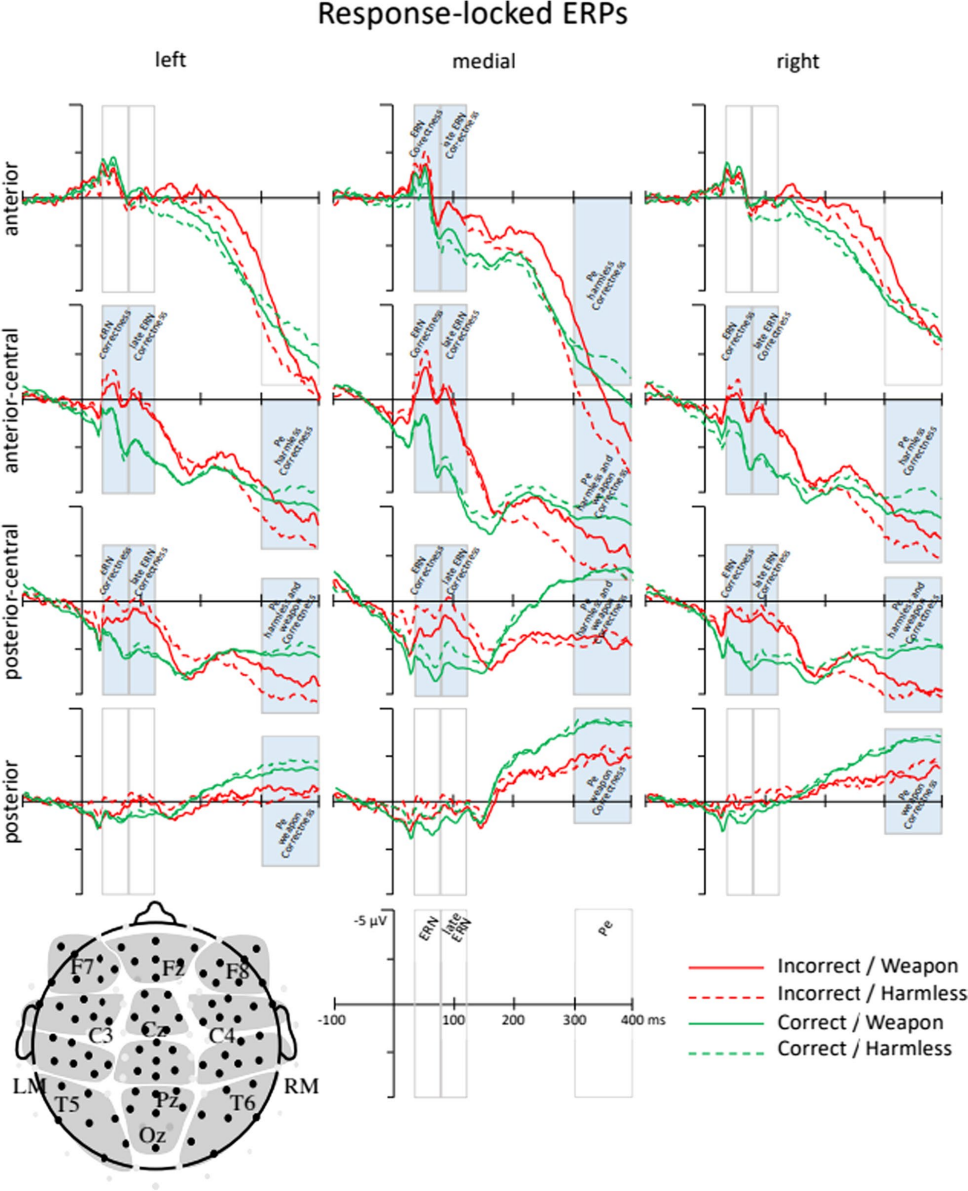
ERP waveforms time-locked to responses. The timing of the error-related component measurement windows (ERN: 25–75 ms; late ERN: 75–125 ms; Pe: 300–400 ms) is indicated with gray rectangle. Correctness is distinguished by line color (incorrect = red, correct = green); Object type is distinguished by line type (weapon = solid, harmless = dashed). Incorrect responses elicited a larger negativity (ERN) and positivity (Pe) than incorrect responses, with the effect of correctness for harmless objects more broadly distributed and larger over some brain regions. Effect significance over each scalp region is indicated by a filled rectangle

A six-way omnibus ANOVA (Emotion × Race × Object × Correctness × LMR × ACP) was run on mean ERP amplitudes 25–75 ms after participants’ responses on the weapon identification task to test for automatic error processing. Consistent with past studies on the ERN (Amodio et al., 2004, 2008), incorrect responses elicited a larger negativity than correct responses (main effect of Correctness: *F*(1, 74) = 57.57, *p* < 0.001, *d* = 0.48) (see Fig. 5). Although the Correctness effect was evident across the entire scalp, a significant Correctness × LMR × ACP interaction (*F*(6, 444) = 26.34, *p* < 0.001, *d* = 0.09) suggested that the strength of the ERN effect differed across electrode positions. Follow-up analyses disaggregated the interaction by anterior-to-posterior scalp regions and found significant Correctness × LMR interactions at anterior (*F*(2, 150) = 35.44, *p* < 0.001, *d* = 0.29), anterior-central (*F*(2, 150) = 31.35, *p* < 0.001, *d* = 0.23), and posterior-central (*F*(2, 150) = 7.05, *p* = 0.001, *d* = 0.11) regions. The Correctness × LMR interactions were followed up by testing for simple effects of Correctness at each scalp location (see Table 6 and Fig. 6).

**Table 6:** Simple effects of correctness on ERN amplitude, as a function of electrode position

	Left		Mid		Right	
	*t*(303)	Effect size	*t*(303)	Effect size	*t*(303)	Effect size
Anterior	− 1.95^†^	− .11	3.89**	.22	− 1.21	− .07
Anterior-central	9.29**	.53	11.84**	.68	10.47**	.60
Posterior-central	8.65**	.49	10.72**	.62	10.68**	.61

Paired sample *t* tests reporting significance and effect size (Cohen's d) of Correctness effects on ERN amplitudes across electrode positions: left, medial, right (LMR) and anterior-to-posterior (ACP)

*p < .05; **p < .001; ^†^*p* < .10

Consistent with past literatures on the ERN, the effect of Correctness was largest over medial anterior-central regions (*t*(303) = 11.84, *p* < 0.001, *d* = 0.68; difference in ERN amplitude: Incorrect − Correct = − 2.74)*.* Because we were interested in whether ERN amplitude sensitivity to errors differed as a function of Emotion, Race, or Object, we conducted a four-way ANOVA (Emotion × Race × Object x Correctness) at the medial anterior-central region where Correctness effect sizes were largest. This analysis did not yield any evidence to suggest that the ERN at medial anterior-central regions was moderated by Race, Object, Emotion, or any interactions among these variables (all interactions involving Correctness × Race, Correctness × Object, Correctness × Emotion and Emotion × Race × Object × Correctness, *p* > 0.50, statistics reported in supplemental information). Response-locked ERP waveforms depicting the effects of Emotion, Race, Object, and Correctness on the ERN measured over medial anterior-central regions can be seen in Fig. 5.

#### Error-related positivity (Pe)

A six-way omnibus ANOVA (Emotion × Race × Object × Correctness × LMR × ACP) was run on mean ERP amplitude 300–400 ms after participants made a response to test for controlled error processing during the weapon identification task. Consistent with existing literature on the Pe (Donchin et al., 1988; Herrmann et al., 2004; Leuthold & Sommer, 1999), a main effect of Correctness reflected larger Pe amplitudes following incorrect, compared to correct, responses (main effect of Correctness: *F*(1, 74) = 9.62, *p* = 0.003, *d* = .19) (see Fig. 6). A significant Object × Correctness × LMR × ACP interaction (*F*(6, 444) = 3.37, *p* = 0.028, *d* = .02) suggested that the effect of Correctness differed by object type across electrode positions. Follow-up analysis disaggregated the interaction by object type and found significant Correctness × LMR × ACP interactions for both harmless objects (*F*(6, 444) = 3.22, *p* = 0.004, *d* = .03) and weapons (*F*(6, 444) = 3.64, *p* = 0.004, *d* = .05), which indicated that the effect of Correctness was generally more posteriorly distributed for weapons than for harmless objects (as shown in Fig. 6), and was medially weighted overall but had increasingly broad lateral distribution with increasingly centroposteriority. This three-way interaction was further disaggregated by anterior-to-posterior scalp regions. For harmless objects, significant Correctness × LMR interactions were found at anterior (*F*(2, 148) = 6.64, *p* = 0.004, *d* = 0.06), anterior-central (*F*(2, 148) = 7.07, *p* = 0.001, *d* = 0.06), and posterior-central (*F*(2, 148) = 5.34, *p* = 0.006, *d* = 0.09) regions. For weapons, significant Correctness × LMR interactions were found at anterior-central (*F*(2, 148) = 5.01, *p* = 0.007, *d* = 0.06), posterior-central (*F*(2, 148) = 23.3, *p* < 0.001, *d* = 0.19), and posterior regions (*F*(2, 148) = 6.10, *p* = 0.004, *d* = 0.09). Significant Correctness × LMR interactions were followed up by testing for simple effects of Correctness at each scalp location (see Table 7).

**Table 7:** Simple effects of correctness on Pe amplitudes, as a function of object and electrode position

	Harmless						Weapon					
	Left		Medial		Right		Left		Medial		Right	
	*t*(151)	*d*	*t*(151)	*d*	*t*(151)	*d*	*t*(151)	*d*	*t*(151)	*d*	*t*(151)	*d*
Anterior	1.17	− 0.10	− 1.98*	− .16	− 1.02	− .08	N.S.	–	N.S.	–	N.S.	–
Anterior-Central	− 2.81*	− .23	− 4.10**	− .33	− 3.77**	− .31	− 0.08	− .07	− 2.34*	− .19	− 1.33	− 0.11
Posterior-Central	− 5.20**	− 0.42	− 7.47**	− .61	− 5.64**	− .46	− 3.33*	− .27	− 8.25**	− .67	− 4.78**	− 0.39
Posterior	N.S.	–	N.S.	–	N.S.	–	− 2.91*	− .24	− 5.82**	− .47	− 5.99**	− 0.49

Paired sample *t* tests reporting significance and effect size (Cohen's d) of Correctness effects on PE amplitudes, as a function of object type (harmless vs. weapon) across electrode positions: left, medial, right (LMR) and anterior-to-posterior (ACP). N.S. indicates that the higher order interactions were non-significant and did not justify *t* tests at a given scalp location

*p < .05; **p < .001

Consistent with prior work, the effects of correctness on Pe were largest over medial anterior-central and medial posterior-central scalp regions (see Table 7). Because we were interested in whether the effects of correctness on Pe amplitude differed as a function of Emotion, Race, or Object, we conducted an Emotion × Race × Object × Correctness ANOVA at medial anterior-central and medial posterior-central regions where Correctness effect sizes were largest. At medial anterior-central regions, this analysis yielded a marginal Object × Correctness interaction (*F*(1, 74) = 3.71, *p* = 0.058, *d* = 0.11). Disaggregating this interaction by Object type found significant effects of Correctness that were larger for harmless objects (Correctness: *t*(151) = − 4.11, *p* < 0.001, *d* = − 0.33; difference in Pe amplitude: Incorrect − Correct = 3.02 μV), compared to weapons (Correctness: *t*(151) = − 2.34, *p* = 0.021, *d* = − 0.19; difference in Pe amplitude: Incorrect − Correct = 1.41 μV). The Object × Correctness interaction was not significant at medial posterior-central regions (*F*(1, 74) = 0.95, *p* = 0.333, *d* = 0.05). Furthermore, there was no evidence to suggest that the Pe was moderated by Race, Emotion, or an Emotion × Race interaction (all interactions involving Correctness × Race, Correctness × Emotion, *p* > 0.25, statistics reported in supplemental information). Response-locked ERP waveforms depicting the effect of Object and Correctness on the Pe can be found in Fig. 6.

## Discussion

The main goal of the present study was to examine the ways in which anger influences the cognitive processing of irrelevant (race) and relevant (object) stimuli during a weapon identification task, and whether it impacts task performance (response times and accuracy). To summarize, our results showed that compared to a neutral state, anger (1) increased N1 amplitudes in response to all faces regardless of race, (2) suppressed racially biased increases in P2 amplitudes in response to Black, compared to White, faces, and (3) suppressed racially biased increases in N2 amplitude in response to White, compared to Black, faces. Based on existing ERP literatures on visual N1, P2, and N2 components, we interpret these findings to suggest that anger (1) increased early automatic attention allocation to all face stimuli even though they were irrelevant to task goals, (2) decreased vigilance specific to Black faces, and (3) decreased cognitive control and inhibition processes specific to White faces. Behavioral results also suggested that anger slowed down overall response times on the task, especially on trials involving the correct identification of harmless objects. Anger, however, did not influence overall accuracy on the weapon identification task, or sensitivity to race or object biased errors as indexed by ERN and Pe. Additionally, the race of primes did not influence task performance or sensitivity to biased errors as indexed by ERN and Pe.

These findings partially support the anger as goal attainment motivation hypothesis (H2), suggesting that anger motivates goal attainment by focusing attentional and memory resources away from task-irrelevant stimuli like race by suppressing preferential sustained attention (vigilance) to Black faces (indexed by P2 amplitudes), and reducing preferential inhibition of White faces (indexed by N2 amplitudes). This was reflected in angry participants’ slower processing of harmless objects and comparable task performance to neutral participants. Less clear, however, is whether the effect of anger on N1 amplitudes, which suggests that anger facilitated automatic attention allocation to task-irrelevant faces (both Black and White), fits with H2. Speculations as to how this seemingly contradictory N1 effect might fit with H2 are explored below.

### Behavioral responses

Consistent with the threat superiority effect, behavioral responses during the weapon identification task demonstrated evidence of weapon bias on both response times and accuracy. As found in previous research on threat superiority, participants were more likely to misidentify harmless objects compared to weapons. They also took longer to correctly identify objects as harmless compared to weapons, presumably because of the effort required to overcome the weapon bias (Blanchette, 2006; Fox et al., 2007; Pratto & John, 1991; Subra et al., 2018).

Interestingly, anger (compared to neutral) exacerbated the slowing of correctly identifying harmless objects compared to weapons, but had no effect on accuracy. A possible explanation for this pattern of results is that angry participants took longer to reach certain decisions during the task because they were making an effort to correct processing errors and weapon biases before they feed forward into behavioral errors. This explanation is consistent with the anger as goal attainment motivation hypothesis (H2), as well as threat superiority literatures suggesting that longer response times involved in correctly identifying harmless objects reflect increased cognitive effort involved in overcoming the threat superiority effect. It also explains why angry participants performed as well as neutral participants in terms of task accuracy.

Despite previous research showing evidence of race bias on the weapon identification task (Amodio et al, 2004, 2008; Payne, 2001), the current study found no evidence to suggest that race influences participants’ behavioral responses in terms of accuracy or response time. We propose two explanations for the failure to replicate race effects on behavior. First, the racial stereotype that drives the weapons bias effect may be a specific association linking Black men to handguns, which may not generalize to Black women or other types of weapons. Indeed, Payne (2001) used male faces only (Black and White) and images of handguns only, whereas we used both female and male faces (Black and White) and images of a wide variety of weapons (handguns, assault rifles, knifes, bombs, and nunchucks; See “Appendix”). These differences in task design may have diluted the impact of race stereotypes on judgments during the weapon identification task. Unfortunately, we are unable to tease apart behavioral task performance or neural processing by separate face primes and object types because of overall low error rates (*M*_errors_ = 18.76). Including prime gender and specific objects would not provide a sufficient number of errors to assess the effects of correctness, which was an important factor for both behavioral and response-locked ERP analyses, and raises concerns about statistical power. However, future studies should examine whether racial stereotypes related to danger uniquely target Black men in the context of particular types of weapons.

An alternate explanation for the failure to replicate past race bias effects on behavior may be the current moment in American history and societal attention to race and racism brought about by the Black Lives Matter movement. In support of that speculation, Sawyer and Gampa (2018) examined Americans’ implicit and explicit racial attitudes using data from the research site called Project Implicit, where people anonymously take Implicit Association Tests assessing their implicit attitudes toward various social groups. Data from over 1.3 million U.S. citizens between January 1st, 2009 and June 30th, 2016, showed that White Americans’ implicit and explicit racial attitudes became less pro-White during a 3-year period after July 6, 2013, during the trial related to the murder of Trayvon Martin, when Black Lives Matter movement gained significant media attention (Sawyer & Gampa, 2018), compared to the 4.5 years before. Because data from the current study was collected after this time period (2016–2018) when attention to racism was prominent in American consciousness as compared to earlier periods when the original weapon identification research was conducted, it is possible that a lack of race bias replication is related to the public consciousness of racism. This explanation is speculative because we did not measure participants’ racial attitudes. Future studies should address this limitation by measuring and controlling for participants’ racial attitudes.

### Attention allocation and response inhibition to task-irrelevant racial cues

The present study also examined the effects of emotion and race on the visual N1, P2, and N2 components elicited by faces prior to behavioral responses. These components have been linked to several cognitive processes across various domains; one prominent interpretation is that N1, P2, and N2 amplitudes are indices of early automatic attention allocation, sustained attention (vigilance), and response inhibition respectively. Several studies provide evidence in support of this interpretation, as discussed in the introduction. That said, we acknowledge that the interpretation of N1, P2, and N2 amplitudes remains suggestive.

#### N1

N1 amplitudes did not significantly differ as a function of race. The absence of a race effect on the N1 is conceptually similar to ERP research by Ito and Urland (2005), who reported inconsistent race effects on N1 amplitudes depending on task complexity. Specifically, they found that the race of faces had no effect on N1 amplitudes when faces were presented as irrelevant stimuli during a complex task. Our weapon identification task was fast moving and complex, and faces were presented as irrelevant to the goals of the task, making it parallel to Ito and Urland’s prior work. Thus, the lack of race effects on N1 amplitudes in this study may be the result of task complexity, such that participants successfully ignored faces in order to meet the task goal of identifying weapons accurately.

N1 amplitudes were however sensitive to emotion, such that angry participants showed larger N1 amplitudes compared to neutral participants. This suggests that anger caused participants to attend more to task-irrelevant face primes. We offer two competing interpretations of this finding in regard to weapon identification task performance and the anger as goal attainment motivation hypothesis (H2). The first is that anger might compromise task performance by facilitating automatic attention allocation to task-irrelevant face stimuli. This would explain why angry participants took longer to correctly identify harmless objects as weapons, as redirecting early attentional resources toward task-irrelevant faces would increase the amount of effort required to overcome the threat superiority effect. It would not, however, explain why overall accuracy was unaffected by anger, nor would it explain the observed P2 and N2 results suggesting that anger suppressed the effects of race on vigilance and inhibition.

A more parsimonious interpretation of the effect of anger on the N1 component would be that increased early attentional allocation to faces among angry (compared to neutral) participants facilitated early disengagement from the task-irrelevant face stimuli, preventing later downstream processing of specific stimulus features (such as race). This would explain why anger seemed to increase early attention without regard to race (N1), but also suppress later race effects on vigilance (P2) and inhibition (N2). Greater early allocation of attention to task stimuli in general (regardless of relevance) could also indicate an increase in overall task engagement among angry participants, which would explain why slower categorization of harmless objects did not come at the cost of task accuracy. While this interpretation of N1 emotion effects is consistent with H2, it remains speculative.

#### P2

Consistent with the anger as goal attainment motivation hypothesis (H2), Black faces elicited larger P2 amplitudes than White faces among neutral, but not angry participants. In line with previous research linking the visually evoked P2 to attention (Anllo-Vento et al., 1998; Hillyard & Münte, 1984; Luck & Hillyard, 1994), the effect of race on P2 amplitudes was largest at medial anterior and medial anterior-central regions. From past research linking larger P2 amplitudes with sustained attention and vigilance to threats (Carretié et al., 2001; Correll et al., 2006; Schutter et al., 2004), we interpret our finding to suggest that neutral participants who showed greater attentional vigilance to Black compared to White faces may have viewed Black faces as more threatening than White faces. Interestingly, anger induction attenuated the P2 sensitivity to racial cues. That is, anger induction reduced threat sensitivity to Black faces, equating them with White faces. This finding is consistent with previous research by Harmon-Jones (2019) that suggests anger motivates goal attainment by suppressing attentional processing of task-irrelevant information. In this case, anger motivated greater accuracy on the weapon identification task by suppressing vigilance racial cues that are task-irrelevant.

#### N2

Black faces elicited smaller N2 amplitudes than White faces. However, the distribution of this race effect on N2 amplitudes across the scalp differed across emotion condition. Specifically, the effect of race was broadly distributed across anterior and anterior-central regions in neutral participants, but was observed only at right anterior-central and right posterior-central regions in angry participants. Because the distribution of the race effect across the scalp is largely consistent with past research observing N2 inhibition effects at frontal (i.e., anterior) regions (Bruin & Wijers, 2002; Pfefferbaum et al., 1985; Jodo & Kayama, 1992; Falkensten et al., 1999), we interpret the race effect on N2 amplitude as reflecting differences in inhibition of Black and White faces. Indeed, among neutral participants the effect of race on the N2 component was shown to be strongest at the medial anterior region, suggesting that they were more likely to engage in inhibitory processes in response to White (compared to Black) faces (also consistent with past research; Correll et al., 2006; Ito & Urland, 2005). Among participants in the anger condition however, the suppressed distribution of race effects to the right anterior-central and right posterior-central regions (as well as smaller race effect sizes at these regions) may suggest that the preferential inhibition of White faces was suppressed among angry participants. As with the P2, we interpret this as further evidence that anger motivated accurate object identification through the suppression of inhibitory processes based on irrelevant racial cues (i.e. White faces), consistent with H2.

### Error processing during the weapon identification task

The current study examined both automatic and controlled error processing by targeting the ERN and Pe respectively as participants engaged in the weapon identification task. Results found a canonical ERN effect, such that incorrect responses on the weapon identification task elicited larger ERN amplitudes compared to correct responses. Consistent with past research, this ERN effect was largest at medial anterior-central regions, supporting extant research suggesting that the ERN propagates from the anterior cingulate cortex. Contrary to our hypothesis, no evidence was found to suggest that ERN amplitudes were sensitive to emotion, race, or object type.

The lack of emotion, race, or object type moderation of ERN amplitudes is especially interesting given past research indicating that automatic error recognition processes are sensitive to racially biased errors on the weapon identification task and associated with higher levels of control (Amodio et al., 2004, 2008). However, it is consistent with the lack of behavioral evidence of racial bias in the present study. One explanation for the failure to replicate both race and object bias when comparing results from the current study to Amodio et al., (2004, 2008) has to do with procedural differences. In Amodio et al., (2004, 2008), participants were explicitly told that the misidentification of a tool as a gun following a Black face prime was “indicative of racial prejudice because it represented an inappropriate application of Black stereotypes”. In contrast, we did not give any explicit directions to suggest that specific errors were tied to race bias or object bias. Differences in these results suggests that automatic error detection processes only detect racially biased errors when the potential for making such errors is made salient to participants.

Despite a lack of ERN sensitivity to race or objects, the Pe effect (indicated by larger positive-going amplitudes after incorrect responses compared to correct responses) was sensitive to object bias. Specifically, a larger Pe effect was detected on trials where harmless objects were misidentified as weapons. This suggests that conscious error detection and re-evaluation processes were particularly sensitive to biases in commission of errors that were consistent with the threat superiority effect. Specifically, participants were most conscious of, and likely to re-evaluate task performance after, misidentifying harmless objects as weapons. That said, despite greater activation of error detection and re-evaluation processes following the misidentification of harmless objects, behavioral responses still indicated significant weapon bias. In other words, awareness of errors consistent with the threat superiority effect was not sufficient to prevent participants’ weapon biases.

## Broader implications

Taken as a whole, our findings suggest that future research aimed at understanding the social cognitive reasons driving costly errors in weapon misidentification should consider the impact that emotional states, such as anger, can have on neurocognitive processes and decision-making. With the exception of the N1, we found evidence supporting the anger as goal attainment motivation hypothesis (H2), such that inducing angry (compared to neutral) emotional states (1) suppressed task-irrelevant race-based vigilance and inhibition processes, (2) increased efforts to overcome the threat superiority effect (indicated by the slower categorization of harmless objects), and (3) led to overall task accuracy comparable to that of neutral participants.

Our work shows that anger does not always increase neurocognitive and behavioral bias. Despite implications that anger can compromise weapon identification task performance by facilitating activation of task-relevant (weapon) and task-irrelevant (race) biases (Baumann & Desteno, 2010, DeSteno et al., 2004; Dasgupta et al., 2009; Bodenhausen et al., 1994), our data suggest that anger may induce goal-oriented behavior that suppresses vigilance and inhibition to task-irrelevant racial cues and motivates slower processing of harmless objects.

Results from the current study indicated that controlled (Pe), but not automatic (ERN), error detection processes were sensitive to errors consistent with threat superiority (i.e., the misidentification of a harmless object as a weapon). It remains unclear whether conscious awareness about biased mistakes (indexed by Pe amplitude) can improve task performance on weapon identification tasks. Future research should further probe the role of conscious error detection processes on biased errors during a weapon identification task. Should future research find an association between Pe amplitude and reduction in race and weapon bias on weapon identification tasks, such a discovery may be a useful foundation to develop training that relies on controlled post-error processing to increase accuracy in threat perception in the lab and in the real world.

Finally, while anger seems to have motivated goal attainment within our lab task, the effect of anger on race and object processing may not generalize to real-world instances of law enforcement officers’ interactions with armed and unarmed suspects for at least three reasons. First, our research was conducted with undergraduate students, not police officers. Second, our research context was tightly controlled and devoid of external stimulation, whereas real-world situations involve more external stimulation and higher levels of negative emotion and stress. Third, in our research, anger was induced by having participants reflect on past autobiographical events unrelated to the weapon identification task, whereas in real world law enforcement situations, anger is likely to be elicited by the law enforcement officers' interactions with the people they suspect. Differences in the source of anger and its relation to the situation at hand is likely to be an important variable that limits generalizability of these findings to real world law enforcement.

## Data Availability

The datasets used and/or analyzed during the current study are available from the corresponding author on request.

## Appendix

Black faces:

White faces:

Weapons:

Harmless objects:

## Abbreviations

EEG: Electroencephalogram
ERP: Event-Related Potential
ERN: Error-Related Negativity
Pe: Error-Related Positivity
LMR: Left, Medial, Right
ACP: Anterior, Central, Posterior

## Footnotes

1 For all ANOVAs, Greenhouse–Geisser corrected results are presented whenever Mauchly’s Test for Sphericity indicated the assumption of sphericity was violated.

2 ERN results are presented for the 25–75 ms time window only. Results were the same in the 75–125 ms time window but are not reported for brevity.

3 Supplemental information can be download with the following OSF link: https://osf.io/dqxtw/?view_only=45d1f1968dd9420c9a084e5212219412.
